# Reasons influencing the nurses’ prioritization process while preventing and managing delirium: findings from a qualitative study

**DOI:** 10.1007/s40520-024-02818-3

**Published:** 2024-08-26

**Authors:** Luisa Sist, Nikita Valentina Ugenti, Stefania Chiappinotto, Rossella Messina, Paola Rucci, Alvisa Palese

**Affiliations:** 1 Department of Biomedical and Neuromotor Sciences, Alma Mater Studiorum University of Bologna, Bologna, Italy; 2 IRCCS Azienda Ospedaliero-Universitaria Di Bologna, Bologna, Italy; 3 Department of Medicine, University of Udine, Udine, Italy; 4 Viale Ercolani, 6, 40138 Bologna, Italy

**Keywords:** Delirium; delirium/nursing; prevention, Management, Prioritisation, Reasons, Qualitative analysis, Thematic Analysis

## Abstract

**Background:**

Nurses play an important role in the prevention and management of delirium episodes. However, some studies have reported that not all interventions recommended are applied due to time and resource constraints, resulting in patients receiving less care than required because other patients and/or interventions are prioritised. The concept of prioritization is part of the broader concept of decision-making as the ability to choose between two or more alternatives to ensure patient safety. Understanding the reasons influencing the prioritization process in patients at risk or with delirium may inform interventions to prevent and/or minimise the unfinished nursing care.

**Aim:**

The purpose of this study was to explore the reasons that inform the prioritisation process among nurses when they are challenged to make decisions for patients at risk and with delirium.

**Methods:**

A descriptive qualitative study performed according to the COnsolidated criteria for Reporting guidelines, in 2021. An intentional sample of nurses working full-time with older patients in medical, geriatric, and post-acute care facilities affiliated with the National Health System was involved. Semi-structured interviews were conducted and narratives thematic analysed.

**Results:**

A total of 56 nurses (55.4% in internal medicine, 26.8% in geriatrics and 17.8% in post-acute/intermediate care) participated with an average age of 31.6 years. The reasons informing the prioritisation process while providing preventive or managerial interventions towards a patient at risk of or with delirium are set at three levels: (1) unit level, as reasons belong to the inadequacy of the ‘Environment’, the ‘Human Resources’, and the ‘Organisation and Work Processes’, (2) nurse’s level, as issues in ‘Competencies’ and ‘Attitudes’ possessed, and (3) patient level, due to the ‘Multidimensional Frailty’.

**Conclusion:**

Nurses caring for patients at risk of and with delirium face several challenges in providing care. To prioritise preventive and managerial interventions, it is essential to implement multilevel and multifaced organizational and educational strategies.

**Supplementary Information:**

The online version contains supplementary material available at 10.1007/s40520-024-02818-3.

## Introduction

Delirium is still a priority problem [1] with a prevalence of approximately 30% in geriatric and internal medicine up to 70% among older residents living in long-term care facilities [2–4]. Among the predisposing factors for delirium, advanced age, cognitive impairment, dementia, and frailty have been underlined; in both hospital and long-term care settings, delirium-related adverse outcomes include decreased independence in activities of daily living and an increased risk of mortality. Consequently, delirium is still a concern for its influence of patient safety, and for its impact on healthcare professionals work processes.

Nurses play an important role in preventing and managing episodes of delirium as underlined by available guidelines [5]; however, as emerged recently [6] several recommendations are not applicable due to time and resources restrains [7], thus causing the patient to receive less care than required because other patients and/or interventions are prioritised.

The concept of prioritisation is part of the broader concept of decision making, defined as the ability to choose between two or more alternatives with the aim of pursuing the goal of patient safety [8]. The need to perform multiple tasks (e.g. administering medications) and the cognitive process (e.g. the knowledge and experience possessed) are combined to optimise the decision-making processes [9]. However, as reported in the literature [10, 11] when nurses establish a sequence of care activities they may decide to delay or omit those perceived as less important [12] generating the so-called Unfinished Nursing Care (UNC) phenomenon describing any nursing interventions needed by the patient/family which is delayed or omitted [11]. The UNC conceptual model has been established as consisting in multilevel elements (i.e., macrosystem, ecosystem, mesosystem, microsystem, and nurse-related level), with antecedents in poor resources and consequences in the poor quality of care [13].

Although the concept of prioritisation is quite new, several studies have established the influencing elements as the patient needs [14]; the context of care (e.g. acute, chronic); [15]; the philosophies, models of care and its organisational aspects [16]; the resources available [16, 17]; and the training; experiences; personalities; values; and beliefs of the nurses [18, 19]. The prioritization may also be associated with the patient’s profile: in the specific context of delirium prevention and management, nurses have been reported to prioritise some interventions as ‘Monitoring the vital parameters (heart rate, blood pressure, oxygen saturation)’ and ‘Ensuring a safe environment (e.g. reducing bed height)’ [7]; on the other hand, they have been documented to rank at low priority the family and/or caregivers involvement and education (e.g., ‘How to re-orient the beloved’) and the presence of clocks, calendars, and specific signs in the room allowing re-orientation [7, 20]. However, the reasons already documented for UNC in general settings (e.g., [21, 22]) have not been integrated with data regarding patients with delirium. Understanding the reasons informing the prioritization process among at-risk and/or patients with delirium can improve clinical outcomes, promote quality improvement strategies and reduce the impact on the individual, his/her relatives, on health care professionals, and ultimately, on the entire organisation [23]. Therefore, the aim of this study was to explore the reasons informing the prioritisation process among nurses while stimulated to make decisions for patients at risk of and with delirium.

## Methods

### Study design

A qualitative study was conducted in 2021 and here reported according to the Consolidated criteria for reporting qualitative research appropriate guidelines [24].

### Setting and participants

The study involved a public research and academic hospital located in Northern Italy, characterised by 49,000 admissions per year and 1,515 beds with a staff of 6807 employees, of which 2478 were nurses. In addition, we involved three private post-acute, extensive and intensive rehabilitation hospitals, with 90 beds affiliated with the health system and equipped with 45 nurses [25]. Specifically, 11 clinical wards (three post-acute and eight academic hospital) with the mission to provide diagnosis, treatment and care of acute and post-acute internal medical patients, were considered as the setting of the study. All wards participated on a voluntary basis: the process of involvement started with the presentation of the project aims to the nurse managers. After having obtained their acceptance, an intentional sample [26] of nurses with the following characteristics were deemed eligible: (a) clinical nurses working full-time in the identified units; (b) able to understand and communicate in Italian; (c) with at least 6 months of clinical experience [9]; and (d) willing to participate in the study. Nurses with organisational roles (e.g. nurse managers) were excluded [27]. Potential participants were invited through a communication from the nurse managers and the researcher (LS) at the shift changes; at the end, 56 nurses provided their contact details to participate.

### Data collection process

The research team (see authors) developed a scenario (Table 1) to stimulate nurses to think and define priorities regarding preventive and managerial interventions needed. The main questions to investigate the reasons influencing the prioritisation were developed according to the available evidence [28] (Table 2). The scenario was provided prior to the meeting, whereas the interview questions were not shared with participants in advance. After obtaining the consent to participate in the study, interviews were scheduled between May and June 2021. All meetings took place online, via the Zoom platform. Each meeting lasted approximately 105 min (range: 90–120 min). 19 meetings were conducted by two researchers (LS, NVU), where the participants ranged from one to seven. The researchers act as observer (NVU) and interviewer (LS), respectively. Audio-visual recordings and in-the field notes were collected to capture all details [29]. The participants were asked to classify the preventive (first sub-scenario) and the managerial (second sub-scenario) interventions by indicating their priorities; for each priority, the underlying reasons were asked, and the reasons freely reported were audio-recorded. A short questionnaire was administered to collect some socio-demographic and professional data.

**Table 1:** The scenario of Mrs. M

Sub-scenario: prevention
Female M aged 84 years, presented to the Emergency Department with dyspnoea, cough and fever for 3 days. Concomitant diseases: Hypertension, COPD and hypercholesterolemia. Home treatment: on amlodipine, ipratropium bromide and simvastatin. In the emergency room she was given intravenous diuretics, steroids, antibiotics and oxygen, and a bladder catheter was placed for fluid monitoring. Prior to admission she lived with her husband, was autonomous in instrumental and basic activities of daily living, drove a car and played cards. After 2 h in the emergency room, she was transferred to the medical unit with the diagnosis of pneumonia. At the nurse’s assessment in the medical unit the following data were noted: TC 38.8 °C, regular HR 70 bpm, BP 140/68 mm Hg, RR 24 beats/min, SpO_2_ 92% with venturi mask FIO2 28%; shallow breathing, presence of productive cough with dense, yellow sputum; no skin turgor; PAINAD 5/10; wearing glasses and hearing aid. On admission, in the morning shift, Mrs. M is unable to answer questions appropriately, shows difficulty in maintaining attention and disorganised thinking, seems to talk to herself and it’s difficult to understand what she says. In addition, she does not know why she is in hospital and thinks it is 1990. Her daughter is worried because she has noticed that her mother is very confused. The following are prescribed: blood cultures, sputum cultures, oxygen therapy with venturi mask FIO2 28%; antibiotic intravenous therapy every 6 h, painkiller, antihypertensive, statins, steroids and diuretics
Sub-scenario: management
At 3 a.m., Mrs M’s daughter called the night nurse because she had psychomotor agitation, had removed her PVC and was trying to get out of bed. Her daughter reported that her mother had been evacuating for the previous 3 days and had refused food and drink for the last 2 days

*BP* blood pressure, *COPD* chronic obstructive pulmonary disease, *FIO*_*2*_ inhaled fraction of oxygen, *HR* heart rate, *RR* respiratory rate, *SpO*_*2*_ oxygen saturation, *PAINAD* Pain Assessment IN Advanced (1–3, mild pain; 4–6, moderate pain; 7–10, severe pain), *PVC* peripheral venous catheter, *TC* body temperature

**Table 2:** Interview guide for clinical nurses: steps and processes

Interview guide
Presentation Aim of the study and data collection process Consent for interview and for audio-recording
**(1) First section**
Sub-scenario regarding the *delirium prevention* Please indicate, in order of priority, the interventions that you will implement to this scenario *Questions*
Sub-scenario regarding the *delirium management* Please indicate, in order of priority, the interventions that you will implement to this scenario *Questions*
**Questions**
‘What are your reasons for making such choices?’
Other questions to clarify or better understand. e.g ‘Why?’ ‘What reasons affect the priorities identified?’ ‘What do you mean?’ ‘Can you explain it a little better?’ ‘What does it mean?’ ‘Can you give examples?’
**(2) Second section**
Completion of the socio-demographic questionnaire via the Wooclap platform (a) Demographic information (age, gender) (b) Undergraduate education (c) Post-graduate education (d) Setting of current work experience (e) Work experience in years

### Data analysis

Quantitative data from the socio-demographic questionnaire was summarised with frequencies, percentages, means and standard deviations/Confidence of Interval (CI; 95%) using Statistical Package for Social Science, version 25. The qualitative data were thematic analysed through the following steps [30].

#### Step 1: Transcription, familiarization with the data, and selection of quotations

One researcher (NVU) transcribed verbatim all the interviews, and a second researcher (LS) checked the accuracy of the transcriptions. Subsequently, three researchers (SC, LS, NVU) independently familiarised themselves with the data by re-reading the transcription several times.

#### Step 2: Selection of keywords

Three researchers (SC; LS; NVU) independently identified the keywords from the text according to their capacity to depict and encapsulate the key concepts as emerging from the transcripts. Then, they compared the key words reaching an agreement.

#### Step 3: Coding

The three researchers (SC; LS; NVU) already involved in the previous steps identified the codes, i.e. short phrases or words explaining the central meaning of the data around the keyword identified.

#### Step 4: Theme development

Then, the same researchers (SC; LS; NVU) started a careful analysis of the identified codes to initiate a detailed interpretation of them and create themes, considering the aims of the study.

#### Step 5: Conceptualization through interpretation of keywords, codes, and themes

The researchers (SC; LS; NVU) completed the conceptualisation phase: the themes were described, and sub-themes identified by checking their consistency with the codes and the keyword selected in the previous steps.

A fourth researcher (AP) was consulted during the process when divergences emerged. With the final step, it was provided the categorization of the themes at different levels by using an inductive and a deductive approach [13].

### Ethical considerations

The study was approved by the Bioethics Committee of the University of Bologna (Italy) register no. 0109186 of 5 May 2021. Participation was voluntary; all nurses gave their written informed consent before being audio recorded and they were allowed to withdraw from the study at any time. In verbatim transcribing the narratives, researchers ensured anonymity by using an alphanumeric code (e.g., RN1); confidentiality was also ensured by anonymising specific details (e.g., the hospital name) encountered during the transcriptions.

### Rigour and truthfulness

According to the available literature [31] to ensure credibility, participants working in the areas of interest were involved; moreover, all researchers (see authors) were experts in the methods and in the topic of investigation. Rigour and reliability were ensured through the following strategies: (a) the use of an interview guide (Table 2); (b) the adoption of a detailed research protocol describing the methodology also regarding the data analysis; (c) a careful collection of the in-the field notes regarding participants’ reasoning, which was shared during data analysis (LS; NVU); (d) the prolonged involvement of several researchers both in the interviews (LS; NVU) and in the data analysis (LS; SC; NVU; AP). Furthermore, an intentional sample was used to ensure transferability, involving nurses caring for patients at risk of or with delirium in medical, geriatric and in post-acute setting.

## Results

### Participants

A total of 56 nurses with a mean age of 31.6 years (CI 95% = 29.6–33.6) participated, of whom 39 (69.6%) were female. Among them, 53 (94.6%) were educated in nursing at the bachelor level, and 12 (21.4%) reported a postgraduate qualification. 15 participants (26.8%) attended also a specific course on delirium.

At the time of the study involvement, 31 (55.4%) nurses were working in internal medicine, 15 (26.8%) in geriatrics and 10 (17.8%) in post-acute care/intermediate care settings. They reported on an average of 4.5 years (CI 95% = 2.7–6.2) of experience and the setting in which they worked at the time of the study was the one in which they had spent most of their professional life (n = 36; 67.9%) (Supplementary Table 1).

### Prioritisation reasons

The reasons informing the prioritisation process in delivering preventive and management interventions towards hospitalised older individuals were identified at three levels: unit, nurse and patient level as reported in Table 3.

**Table 3:** Reasons informing priorities in preventive and managerial interventions in patients at risk and/or with delirium: levels, themes, subthemes, and quotations

Level	Themes	Subthemes	Interventions		Quotations P, preventive M, managerial
			Preventive	Managerial	
Unit Level	Inappropriate care environment		*	*	P ‘Then we don’t have dedicated environments for these types of patients …for example a delirium room, single rooms just like a dedicated environment.… it’s very difficult to manage these patients if you don’t have dedicated environments…’(RN40) ‘…Also the lack of dedicated tools to prevent disorientation, like clocks or calendars to help people understand where they are and what time it is so they don’t get disoriented.’ (RN14)
					M ‘…important to have a dedicated room like the delirium room. To care for a much more cognitively complex patient, they need a dedicated room close to the ward room.’(RN46) ‘…there are no calendars, there are no clocks, there are no forms of entertainment…’ (RN28)
	Inadequate Human Resources	Inadequate nurse/patient ratio	*	*	P ‘…the shortage of staff, because the adequate nurse/patient ratio also allows me to give him a shave, which may be a “superficial” thing, but for an elderly person who has no one, this could make his day. It could also change his approach to therapy…’ (RN42)
					M ‘…We could act in a thousand other ways, but we lack the resources, we have very complex patients and minimal resources, such a situation is not easy to manage to guarantee a minimum level of care…’ (RN46)
		Inadequate nursing aides/patient ratio		*	M ‘the nursing aides are an integral part but there is not even one in 44 patients.’ (RN35)
	Issues in the organisation and work processes	Mission of the ward	*	*	P ‘…I instinctively came to reason as we do in the ward with patients who have problems of this kind…So I tried to focus mainly on priority interventions, those that should be done immediately to prevent or manage a delirium episode…’ (RN43)
					M ‘…I looked at the scenario in the ward where I work…. I in my ward I am really alone…’(RN4)
		Ineffective routines	*	*	P ‘…guided not only by theory, but also by what is the reality of my daily practice…’ (RN18)
					M ‘…I have always drawn on clinical practice and everyday life…’ (RN43)
		Inadequate collaboration with other professionals	*	*	P ‘…I still work in a team and there is one thing I would instinctively say…For example, I don’t do it, the doctor does it…. Or other professionals …. In terms of how I work, the line is very blurred. The aspect of working in a team is definitely a priority…’ (RN1)
					M ‘…managing the patient with delirium within the team…’(RN29)
		Lack of shared documents (tools/procedure/protocols/guidelines)	*	*	P ‘…we never make assessments through scales of risk of delirium and with the presence of delirium and we do not have the tools for assessment…we usually assess whether the person is oriented, disoriented, oriented in time and space, we make assessments but not objective ones…’ (RN2)
					M ‘…If we have a cardiac arrest, we know what to do, i.e. we rely on standardised guidelines. I know that if I do this procedure I will get this result. On the other hand, in the case of delirium or a patient at risk of delirium, I don’t have much material, I don’t have procedures, guidelines, let’s say it’s a bit of a grey area, quote unquote, where I don’t have many elements to refer to…’ (RN20)
		Lack of care continuity		*	M ‘… We pass them on, but it happens that some information is omitted, they get lost, something is neglected, we are not infallible, maybe also because I do not follow them all the time…’(RN40)
		Night shifts challenges		*	M ‘… It’s night, so it’s really a different situation and even more complicated, patients generally decompensate at night, it’s easier for them to get confused and so on and the management is more difficult…’ (RN13) ‘… Here at night you have more time for individual care. Why should I not give her an enema or change a bladder catheter or give her chamomile tea…’(RN42)
Nurses Level	Competencies	The value of the contextual professional experience	*	*	P ‘…I think it guided the experience. I had a type of patient, or more than one patient on my mind, guiding me…’ (RN5)
					M ‘…experience helps, but it’s not necessarily true that someone who’s been working for a short time is going to act wrongly compared to someone who’s been working for many years…’ (RN32)
		Issues in the knowledge about Delirium	*	*	P ‘…I honestly don’t have any knowledge about delirium…I haven’t done any courses and at university we’ve had very little to do with it… So I don’t have any theoretical knowledge about managing the patient at risk of or with delirium…’ (RN40)
					M ‘…We are professionals, so we should also be able to assess according to our experience, skills and training…’ (RN9) M ‘…the priority is also based on knowing the patient and on continuity. It’s logical that it changes, if I see him for the first time and not a colleague who is with this patient and has already known him, for example, for 3 weeks of the patient’s stay, this is very important also to build the relationship of trust that is inevitably created between patient and nurse, patient and doctor, patient and nursing aides…’ (RN40)
		The role of the constant awareness and reflexivity	*	*	P ‘…I have concentrated on the assessment of risk factors for delirium; to identify and treat the possible risk factors for delirium. The lady has various risk factors, so go and intervene on them immediately so that they do not become causes of delirium…’ (RN2) ‘…It’s very important to encourage the person to drink, because of course if the person doesn’t drink they will become dehydrated and that can lead to infection and then disorientation…’ (RN5) ‘…Pain is very important, I put it as a priority because very often people can’t express what they have…’ (RN5) ‘…encourage sleep, bad sleep is going to change the next day’s activities anyway, it worsens the cognitive state of the patients…’ (RN7) ‘…also constipation for example, very often people who have not evacuated for a long time start to become very nervous, they show confusion…’ (RN5)
					M ‘…Assess the risk factors that led to the restlessness, understand why the person had this change…’ (RN5) ‘…as it is 03:00 in the morning, I have included among the priority interventions those that assess sleep activity and promote it… elements that could disturb it…’ (RN2) ‘…I have also given importance to the evaluation of the prevention of changes in intestinal elimination…’ (RN18) ‘…I would have invited her and I would have offered her, I don’t know, some tea instead of some water and I would have made her go into the room…’ (RN26) ‘…patient is confused so she is not able to express the pain, my attention is also focused on the pain by assessing it through the scale and finally treating the pain…’ (RN16)
		Communication abilities	*	*	P ‘…We try to talk, let them express their thoughts…’ (RN52)
					M ‘… I concentrated on what to do first to calm the patient down. Right now the patient is agitated and my thought is to communicate with her, to try to calm her down, to make her understand where she is, to assess her state of agitation through communication…’ (RN2)
		Time management skills	*	*	P ‘…I have concentrated on what you should try to do in the first few hours, then the other interventions are postponed to a later time…’ (RN43) ‘…I prioritised according to a temporal moment…’ (RN25)
					M ‘…Unfortunately, sometimes you realise that there are many things that cannot be done because of lack of time. …With this type of patient…you should have a little more personal support, but you can’t because you have so many things to do during a shift…’ (RN11)
	Attitudes	Being challenged by decisions	*	*	P ‘…so setting the priority and the hardest thing to do, I felt like I was betraying my ideals by putting some things aside…maybe because in practice the distinction is not so clear…like to say maybe because now you think with a cool mind…’ (RN1)
					M ‘…I hate making these decisions. Eh, but it can still be an important one…’ (RN42)
		Living in hurry	*		P ‘…a nurse who is in a hurry and a nurse who does not give her best to the patient and to the patient…’ (RN42)
		Being able to do things simultaneously	*	*	P ‘…There are many interventions that we do in practice at the same time… For example, while I am giving the therapy, I am trying to talk to her to calm her down and give her some instructions… That’s the point of doing things at the same time. I have to rationalise every moment…’ (RN26)
					M ‘…because you can’t choose, that is, it should be one, some things overlap with others…for example, the presence of the family member overlaps with the education of the family member…In my opinion, many activities can be done in an integrated way, none of it is separate, everything can be integrated safely…’ (RN42)
		Shaping priorities around Safety for all as first, Basic needs as first, or Prescriptions as first	*	*	*Safety as first* P ‘…to ensure the safety, especially of the person who is at risk of delirium, because they cannot see where they are hurting themselves and we have to prevent them from hurting themselves…’ (RN46)
					M ‘…That of reassuring the patient, avoiding all interventions of restraint. I look first for other ways, other solutions…’ (RN23) ‘…The effect of restraint always depends on the case, because maybe there are people who are restrained, they get more agitated and maybe by not being restrained they calm down. It has happened that agitated patients have calmed down with restraints and they don’t try to climb over the rails…’ (RN56) ‘…First of all, the safety of the person and to prevent them from wandering off or hurting themselves…’ (RN46) ‘…So the choice also goes on whether you have more than one patient like that… Not just one patient, but also the priority of other patients…’ (RN39) ‘…I also have to be safe while the patient is agitated…’ (RN19)
			*	*	*Basic needs as first* P ‘…Having done that, I would tailor interventions according to the person’s needs…’ (RN29) M ‘…The patient has needs now, needs that are present even if they are not expressed. I focus on the needs that the patient has in this moment of delirium, for example the need to sleep…’ (RN48)
				*	*Prescriptions orders as first* M ‘…Autonomy and also the ability to respond promptly and correctly to what the doctor tells you and asks you. I am the one who assesses the situation and intervenes…’ (RN34)
Patient Levels	Multidimensional frailty	Unavailable caregivers/relatives	*	*	P ‘…the person’s autonomy must be maintained as much as possible to avoid decompensation again so the nutritional intake must be … assessed.’ (RN33)
					M ‘…I prefer the presence of family members when we could and when we can…. It’s hard, hard for patients not to see their children, people get disorientated and even more so without their loved ones.. I have often found patients in a state of confusion…’ (RN24)
		Other competitive clinical issues	*	*	P ‘…I try to stabilise the patient first… there is a possibility of sudden deterioration…’ (RN24) You stabilise the patient first…’ (RN5)
					M ‘…More critical, that is for this type of patient, so here let’s say we had little information, but I was guided by the fact that, that is, it was an acute event, so that is the lady was agitated, so she took off the CVP, tried to get out of bed, so I, that is, I left the clinical aspect alone…’ (RN20)
		Challenges in the needs assessment due to the cognitive state	*	*	P ‘…I chose the second one taking into account the cognitive state, at risk of delirium…’ (RN35)
					M ‘…Because we say that we are dealing with psychomotor agitation of the patient in progress …’ (RN19)

*RN* registered nurses, *P* preventive interventions, *M* managerial intervention, *n* number of interview

* means the presence of preventive/management interventions

### Unit level: issues in the care environment, in the human resources, and in the organization and work process

This level provides the reasons for prioritisation linked to the context in which the patient at risk of and with delirium is admitted and cared for. Prioritization can be influence by the environment, the human resources, and the organization/work processes.

Inappropriate and chaotic care units with several patients in small rooms, with the equipment and the required material to deliver nursing care stored away from the rooms affect the time and the concentration require to undertake the right priorities. Dedicated, safe environments, without architectural barriers and tools (e.g., clock and calendar or with a delirium room), are all limited or absent, thus influencing the prioritization of all space–time reorientation preventive management interventions.

Furthermore, the lack of human resources in terms of nurse-to-patients and nurse aides-to-patient’s ratios forces to take care of those needs perceived as most important, urgent, or critical, leaving others unmet. Moreover, while shortages in nurses affect both preventive and managerial interventions, the shortages in nurses-aides influence only the management of the delirium but not its prevention.

A role is played also by the organisation and work processes: the geriatric mission of the unit increases the attention of nurses towards delirium prevention and management, whereas work processes based on strong routines, i.e. ‘it has always been done this way’, prevent the prioritization of some individual needs, given that all interventions are provided in an established order along the time and the sequence. The poor interprofessional collaboration increases the need to spend time in searching for, discussing, and in communicating with other professionals, thus further reducing the time available for patients. In this context, the lack of specific supportive tools (procedures, guidelines) in the field of delirium prevention and management threatens an effective care delivery, increasing the repetition of some well-acknowledged routinised activities (e.g., evaluating the risk factors), and implicitly delegating the interventions to other professions. In addition, the shift work, where subsequent nurses are involved in the 24/24 care of patients with no specific point of reference as a primary nurse, increases the need to collect data, searching for information regarding what has been done in the previous shifts with discontinuous care delivered to patients based on different priorities. Moreover, although at night nurses have more time to devote to the patient by autonomously organising the work processes, the lack of resources (e.g. two nurses on average for 40 patients) influences the prioritization of the interventions, providing them to urgent/clinical instable patients, leading to UNC.

### Nurse level: the role of the nurses’ competencies and attitudes

Identifying, recognising and managing the predisposing and precipitating factors of delirium require competencies that may influence the priorities. Possessing a specific professional experience in the context of patients with delirium supports nurses in the prioritization process as well as in the early identification of the risks. On the other hand, the lack of knowledge leads nurses to prioritise according to what they have learnt during under and post-graduate education, planning some unnecessary interventions (e.g. monitoring vital signs) and leaving those required neglected. Moreover, nurses set priorities according to their continuous awareness of the situation, and reflexivity of what is going on, where the ability to assess the risks, to perceive them and to hypothesise the patient’ trajectory, anticipating the course of the events may inform the decision of priorities. Nurses’ communication abilities also play a role in detecting patients’ needs when not verbally communicated, which helps in prioritising those not immediate visible.

Time management skills are reported by nurses as another reason influencing the priorities: they emphasise the importance of being able to organise the shift and save time to provide individualised interventions.

Also attitudes were recognised as influencing the priority process. Making decisions may be challenging for nurses; not all could face these challenges and to identify what to put aside, when not everything can be done. Moreover, some nurses live in a ‘hurry’ also when there is no time pressure, as a sort of shaped attitude, reducing the time to invest in the patient care. Additionally, not all nurses are able to do several things simultaneously to optimise time, by overlapping different activities to deliver at the same time, such as communicational-relational and technical interventions, e.g. assessing the risk while taking vital signs. This further reduces the likelihood to prioritise patients at risk of or with delirium.

In setting priorities, nurses follow different schemes, as safety first, needs first, or prescription first, and these different tendencies shaped during education and experience may prevent a common action. The safety approach is not only focused on that of all patients but also on the health care professionals, to prevent legal implications.

### Patient level: the role of the multidimensional frailty

The multidimensional frailty of the patient, characterised by the absence of the carers, the presence of several clinical issues, and the underlying cognitive impairments, has been reported as influencing the prioritization in both preventive and management interventions. The absence of caregivers, due to the restrictive policies introduced during the pandemic, require nurses to spend more time reassuring and staying close to patients, by also replacing family members in performing some tasks (e.g., watching out for falls or supervising them when they become agitated). Moreover, high priority is given to the clinical issues as the critical condition/gravity in the context of all patients, not only towards those at risk of and with delirium; the latter have been underlined as more demanding, especially those with psychomotor agitation, consequently reducing the nurses’ surveillance of stable patients. Patients’ cognitive impairments also influence prioritisation, as nurses find themselves spending more time to establish a trusting relationship with the patient, to understand his/her needs and to manage them.

## Discussion

To the best of our knowledge, this is the first qualitative study based on scenarios involving expert nurses caring for older patients, with different professional and educational backgrounds to discover reasons informing the process of prioritization in hospitalised older patients at risk of and with delirium. The several reasons emerged, identified at three levels, unit, nurses, and patients, suggest the presence of multifaced factors, interacting each other, that should be considered in designing interventions to promote the best quality of care.

First, priorities in these patients are influenced at different levels of the system as documented in the context of UNC for general patients [21, 22]; however, some reasons seem to be specific for patients at risk of or with delirium. Specifically, the inappropriate care environment has been emphasised, but its change is out of the scope of the profession, leaving nurses aware regarding the issues, but ineffective. Programmes, such as the Delirium Room model [32] involving structural, environmental (e.g. lighting) and reorientation tools (e.g. calendar, clock) reduce the duration of delirium [33] but their development is under the responsibility of the hospital. Moreover, nurses have mentioned these reasons without any connection with the scenario administered, suggesting that, also in simulated circumstances, they set priorities as they are used to in the real context, which might highlight the challenges lived by them daily, as well as the barriers that may be encountered in any attempt to change priorities when the environment remains unchanged.

On the other hand, issues in human resources of both nurses and nursing aides [34] have also been mentioned—and further affect the care given to patients at risk of and with delirium who require more time to be understood [35] and managed. Patients with delirium increase the workloads of nurses, thus further limiting the time available; also in this case, reasons influencing priorities are out of the responsibility of the nurses. The additional reasons as the need of integration with other healthcare professionals to provide personalised care have been already underlined [36]: however, the lack of time available prevents multiprofessional initiatives, forcing nurses to work alone to save time. On the other hand, some reasons characterising the work and organisation processes, such as the routinised approaches, the lack of tools for delirium assessment and management and the poor continuity across shifts, which may all affect the early identification and the following consistent management of patients with delirium, are under the responsibility of nurses. During night shifts the UNC may increase also in these patients because the lack of human resources. However, nights are seen by our nurses as an occasion to spend more time outside of routine to deliver personalised care, as already documented [37].

Nurses’ competences and attitudes also play an important role in the delirium prevention and management: nurses act in coherence with their experience and education [38], and their physical and psychological exhaustion [39] may increase the difficulties in making decisions, leading to routinised interventions. Except for some aspects already documented in the literature (e.g. multitasking, [38]), the different priorities are set around different patterns based upon the safety, the needs and the medical prescriptions: these different patterns of actions, when inconsistent across shifts and nurses, may increase uncertainty in the care of patients at risk of delirium. Nurses have been recognised as important in promptly identifying risks and interventions [5] and should be supported by specific tools (e.g., [40]) for identifying, recognising and managing predisposing and precipitating factors for delirium. As in other settings, awareness and reflexivity are the basis of prioritisation and is influenced by the experience acquired in the specific field [41]; however, recognising risks may be difficult when nurses are not supported with appropriate evidence [42]. Therefore, reasons influencing prioritization seems to stimulate both intuitive and analytic reasons processes: the former, when nurses lack in the knowledge, in tools, and in protocols regarding how to manage delirium; the latter, when the risk is assessed according to the experience and knowledge helping in recognising and managing the factors. Therefore, a multilevel strategy, based on mentorship and educational programmes shaping attitudes are needed, for example by identifying expert nurses in delirium prevention and management at the unit level to coach the capacity to identify right priorities and to prevent UNC; undergraduate education also play an important role, as well as postgraduate courses to stimulate continuous awareness and a more consistent and updated ‘delirium literacy’ among nurses [43].

Finally, the multidimensional frailty of patients seems to have a catastrophic effect on prioritization. Patients with other clinical issues are prioritised, suggesting that delirium is not considered as a relevant clinical condition; moreover, the time required to understand the patients’ needs, which may be difficult to identify, is not available: consequently, needs are left unmet. The absence of relatives at the bedside further increases the challenges: family members have already been reported as safe keepers and as a source of additional surveillance of patients [44]. Therefore, patients without relatives should be further considered at the top of priorities to prevent any form of UNC.

Overall, according to the continuum theory model [38], the time available influences priorities, both because the scarcity of time stimulates the identification of some priorities, and because the care and management of patients with delirium requires time to assess their needs and build a trusting relationship. In this context, the silent and imperceptible risk of delirium leads nurses to postpone or miss some interventions, whereas the explosion of delirium requires immediate prioritization of these patients. Moreover, some reasons affect only the prevention (e.g., living in a hurry), while others only the management phase (e.g., night shifts); however, as most reasons are common to both the prevention and the management, suggesting that a comprehensive strategy may ameliorate the whole prioritization process.

### Limitations

This study has several limitations. First, it was conducted during the pandemic and this may have influenced the results given that the process of care and the settings were subjected to several changes; second, there were involved nurses working in medical, geriatric and post-acute settings suggesting that future studies are need to accumulated evidence both in the Italian context (e.g. surgical units) and at the international levels in different health care systems and cultures. Third, the scenario-based data collection process may have prevented the full identification of the reasons influencing the prioritization occurring at the patient’s bedside. Fourth, the data analysis involved an interactive and reflexive process, engaging researchers for long time and involving them in all stages. However, thesubjectivity of researchers and the different familiarity with the thematic analysis adopted may have influenced the findings [29].

## Conclusions

Nurses are used to prioritising interventions; however, while the reasons influencing the process among hospitalised patients has been investigated to detect why nurses unfinish some activities in favour of others, in the context of older people at risk or with delirium, no data have been collected to date. To the best of our knowledge, this is the first study attempting to identify reasons affecting the prioritization process among these patients. Findings suggest that the process is influenced by reasons set at three levels, some of which are under the nurses’ control while others are not, that mainly affect both preventive and managerial interventions.

To promote the right identification of the priorities that may protect older patients from an escalation towards delirium, targeting the (a) resources available at the unit level, the (b) nurses’ competence and attitudes and the (c) patient’ complexity is crucial. Changes in the environment, and ensuring an effective work and organisation processes through collaboration and integration between professionals, by also providing decision-making support tools, are recommended. In addition, given that the mission of the units, as the devoted to geriatric one seems to influence the prioritization, investments towards nurses working in other settings, are important. Moreover, nurses should be educated and supported in developing competencies and attitudes not only during undergraduate but also in postgraduate and continuing education to promote and update their ‘delirium literacy’.

Patients at risk of with delirium play also a role in the prioritization process: they are at risk to be neglected in the beginning because their risks are invisible; later they are still at risk to be receive unfinished care because the increased needs cannot be managed in the context of the care that all patients required. Continuing to investigate the underlying reasons of prioritization is important to accumulate evidence and inform strategies to prevent any form of unfinished care.

## Supplementary Information

Below is the link to the electronic supplementary material.

Supplementary file1 (DOCX 21 KB)

## Data Availability

No datasets were generated or analysed during the current study.

## References

[1] NíChróinín D, Alexandrou E, Frost SA (2023) Delirium in the intensive care unit and its importance in the post-operative context: a review. Front Med 10:1071854. 10.3389/fmed.2023.1071854 10.3389/fmed.2023.1071854 PMC10098316 37064025

[2] Inouye SK, Westendorp RG, Saczynski JS (2014) Delirium in elderly people. Lancet 383:911–922. 10.1016/S0140-6736(13)60688-1 23992774 10.1016/S0140-6736(13)60688-1 PMC4120864

[3] Bellelli G, Morandi A, Di Santo SG et al (2016) “Delirium Day”: a nationwide point prevalence study of delirium in older hospitalized patients using an easy standardized diagnostic tool. BMC 14:106. 10.1186/s12916-016-0649-8 10.1186/s12916-016-0649-8 PMC4950237 27430902

[4] Morandi A, Di Santo SG, Zambon A et al (2019) Delirium, dementia, and in-hospital mortality: the results from the Italian delirium day 2016, a national multicenter study. J Gerontol A Biol Sci Med Sci 74:910–916. 10.1093/gerona/gly154 29982365 10.1093/gerona/gly154

[5] Hoch J, Bauer JM, Bizer M et al (2022) Nurses’ competence in recognition and management of delirium in older patients: development and piloting of a self-assessment tool. BMC Geriatr 22:879. 10.1186/s12877-022-03573-8 36402941 10.1186/s12877-022-03573-8 PMC9675220

[6] Sist L, Ugenti NV, Donati G et al (2022) Applicability of the interventions recommended for patients at risk or with delirium in medical and post-acute settings: a systematic review and a Nominal Group Technique study. Aging Clin Exp Res 34:1781–1791. 10.1007/s40520-022-02127-7 35451735 10.1007/s40520-022-02127-7

[7] Sist L, Pezzolati M, Ugenti NV et al (2024) Nurses prioritization processes to prevent delirium in patients at risk: findings from a Q-methodology study. Geriatr Nurs 58:59–68. 10.1016/j.gerinurse.2024.05.002 38762972 10.1016/j.gerinurse.2024.05.002

[8] Hendry C, Walker A (2004) Priority setting in clinical nursing practice: literature review. J Adv Nurs 47:427–436. 10.1111/j.1365-2648.2004.03120.x 15271162 10.1111/j.1365-2648.2004.03120.x

[9] Bjørk IT, Hamilton GA (2011) Clinical decision making of nurses working in hospital settings. Nurs Res Pract 2011:524918. 10.1155/2011/524918 21994830 10.1155/2011/524918 PMC3182333

[10] Sist L, Palese A (2020) Le decisioni infermieristiche e le missed nursing care: risultati di una scoping review [Decision making process and missed nursing care: findings from a scoping review]. Assist Inferm Ric 39:188–200. 10.1702/3508.34952 33362189 10.1702/3508.34952

[11] Jones TL, Hamilton P, Murry N (2015) Unfinished nursing care, missed care, and implicitly rationed care: state of the science review. Int J Nurs Stud 52:1121–1137. 10.1016/j.ijnurstu.2015.02.012 25794946 10.1016/j.ijnurstu.2015.02.012

[12] Kalisch BJ, Landstrom GL, Hinshaw AS (2009) Missed nursing care: a concept analysis. J Adv Nurs 65:1509–1517. 10.1111/j.1365-2648.2009.05027.x 19456994 10.1111/j.1365-2648.2009.05027.x

[13] Jones T, Willis E, Amorim-Lopes M et al (2019) Advancing the science of unfinished nursing care: exploring the benefits of cross-disciplinary knowledge exchange, knowledge integration and transdisciplinarity. J Adv Nurs. 75:905–917. 10.1111/jan.13948 30644130 10.1111/jan.13948

[14] Akishita M, Ishii S, Kojima T et al (2013) Priorities of health care outcomes for the elderly. J Am Med Dir Assoc 14:479–484. 10.1016/j.jamda.2013.01.009 23415841 10.1016/j.jamda.2013.01.009

[15] Knopp-Sihota JA, Niehaus L, Squires JE et al (2015) Factors associated with rushed and missed resident care in western Canadian nursing homes: a cross-sectional survey of health care aides. J Clin Nurs 24:2815–2825. 10.1111/jocn.12887 26177787 10.1111/jocn.12887

[16] Mandal L, Seethalakshmi A, Rajendrababu A (2020) Rationing of nursing care, a deviation from holistic nursing: a systematic review. Nurs Philos 21:e12257. 10.1111/nup.12257 31429179 10.1111/nup.12257

[17] Ludlow K, Churruca K, Mumford V et al (2020) Staff members’ prioritisation of care in residential aged care facilities: a Q methodology study. BMC Health Serv Res 20:423. 10.1186/s12913-020-05127-3 32410685 10.1186/s12913-020-05127-3 PMC7222492

[18] Drach-Zahavy A, Srulovici E (2019) The personality profile of the accountable nurse and missed nursing care. J Adv Nurs 75:368–379. 10.1111/jan.13849 30209825 10.1111/jan.13849

[19] Ludlow K, Churruca K, Mumford V et al (2021) Unfinished care in residential aged care facilities: an integrative review. Gerontologist 61:e61–e74. 10.1093/geront/gnz145 31773131 10.1093/geront/gnz145

[20] Vreeswijk R, Maier AB, Kalisvaart KJ (2022) Recipe for primary prevention of delirium in hospitalized older patients. Aging Clin Exp Res 34:2927–2944. 10.1007/s40520-022-02249-y 36131074 10.1007/s40520-022-02249-y

[21] Sist L, Chiappinotto S, Messina R et al (2024) The reasons for unfinished nursing care during the COVID-19 pandemic: an integrative review. Nurs Rep 14:753–766. 10.3390/nursrep14020058 38651470 10.3390/nursrep14020058 PMC11036286

[22] Chiappinotto S, Bayram A, Grassetti L et al (2023) Were the unfinished nursing care occurrence, reasons, and consequences different between COVID-19 and non-COVID-19 patients? A systematic review. BMC Nurs 22:341. 10.1186/s12912-023-01513-4 37759199 10.1186/s12912-023-01513-4 PMC10523650

[23] Kinchin I, Mitchell E, Agar M et al (2021) Economic cost of delirium: a systematic review and quality assessment. Alzheimers Dement 17:1026–1041. 10.1002/alz.12262 33480183 10.1002/alz.12262

[24] Buus N, Perron A (2020) The quality of quality criteria: replicating the development of the consolidated criteria for reporting qualitative research (COREQ). Int J Nurs Stud 102:103452. 10.1016/j.ijnurstu.2019.103452 31726311 10.1016/j.ijnurstu.2019.103452

[25] Regione Emilia Romagna (2022) Dati sui servizi nel biennio 2020–2021 a cura dell’Assesorato regionale alle politiche per la salute. https://salute.regione.emilia-romagna.it. Accessed 31 May 2024

[26] Palinkas LA, Horwitz SM, Green CA et al (2015) Purposeful sampling for qualitative data collection and analysis in mixed method implementation research. Adm Policy Ment Health 42:533–544. 10.1007/s10488-013-0528-y 24193818 10.1007/s10488-013-0528-y PMC4012002

[27] Palese A, Bottega M, Cescutti A et al (2020) Depicting clinical nurses’ priority perspectives leading to unfinished nursing care: a pilot Q methodology study. J Nurs Manag 28:2146–2156. 10.1111/jonm.13036 32335959 10.1111/jonm.13036

[28] Bassi E, Tartaglini D, Valpiani G et al (2020) Unfinished nursing care survey: a development and validation study. J Nurs Manag 28:2061–2071. 10.1111/jonm.13170 32985010 10.1111/jonm.13170

[29] Braun V, Clarke V (2006) Using thematic analysis in psychology. Qual Res Psychol 3:77–101 10.1191/1478088706qp063oa

[30] Braun V, Clarke V (2022) Conceptual and design thinking for thematic analysis. Qual Psychol 9:3–26. 10.1037/qup0000196 10.1037/qup0000196

[31] Thomas E, Magilvy JK (2011) Qualitative rigor or research validity in qualitative research. J Spec Pediatr Nurs 16:151–155. 10.1111/j.1744-6155.2011.00283.x 21439005 10.1111/j.1744-6155.2011.00283.x

[32] Flaherty JH, Tariq SH, Raghavan S et al (2003) A model for managing delirious older inpatients. J Am Geriatr Soc 51:1031–1035. 10.1046/j.1365-2389.2003.51320.x 12834527 10.1046/j.1365-2389.2003.51320.x

[33] Lee HJ, Jung YJ, Choi NJ et al (2023) The effects of environmental interventions for delirium in critically ill surgical patients. Acute Crit Care 38:479–487. 10.4266/acc.2023.00990 38052513 10.4266/acc.2023.00990 PMC10718493

[34] Fanton E, Tasca T, Costa C et al (2023) The education of specialized nurses’ aides: current scenario and future perspectives. Assist Inferm Ric 42:218–234. 10.1702/4178.41687 38230556 10.1702/4178.41687

[35] El Hussein M, Hirst S, Salyers V (2015) Factors that contribute to underrecognition of delirium by registered nurses in acute care settings: a scoping review of the literature to explain this phenomenon. J Clin Nurs 24:906–915. 10.1111/jocn.12693 25293502 10.1111/jocn.12693

[36] Kim Y, Lee MJ, Choi M et al (2023) Exploring nurses’ multitasking in clinical settings using a multimethod study. Sci Rep 13:5704. 10.1038/s41598-023-32350-9 37029189 10.1038/s41598-023-32350-9 PMC10082008

[37] Kristiansen S, Konradsen H, Beck M (2019) Nurses’ experiences of caring for older patients afflicted by delirium in a neurological department. J Clin Nurs 28:920–930. 10.1111/jocn.14709 30376210 10.1111/jocn.14709

[38] Thomas N, Coleman M, Terry D (2021) Nurses’ experience of caring for patients with delirium: systematic review and qualitative evidence synthesis. Nurs Rep 11:164–174. 10.3390/nursrep11010016 34968321 10.3390/nursrep11010016 PMC8608072

[39] Teece A, Baker J, Smith H (2022) Understanding the decision-making of critical care nurses when restraining a patient with psychomotor agitation secondary to hyperactive delirium: a ‘Think Aloud’ study. J Clin Nurs 31:121–133. 10.1111/jocn.15889 34056784 10.1111/jocn.15889

[40] Pilotto A, Aprile PL, Veronese N et al (2024) The Italian guideline on comprehensive geriatric assessment (CGA) for the older persons: a collaborative work of 25 Italian Scientific Societies and the National Institute of Health. Aging Clin Exp Res 36:121. 10.1007/s40520-024-02772-0 38797797 10.1007/s40520-024-02772-0 PMC11128394

[41] Melin-Johansson C, Palmqvist R, Rönnberg L (2017) Clinical intuition in the nursing process and decision-making—a mixed-studies review. J Clin Nurs 26:3936–3949. 10.1111/jocn.13814 28329439 10.1111/jocn.13814

[42] Agar M, Draper B, Phillips PA et al (2012) Making decisions about delirium: a qualitative comparison of decision making between nurses working in palliative care, aged care, aged care psychiatry, and oncology. Palliat Med 26:887–896. 10.1177/0269216311419884 21908522 10.1177/0269216311419884

[43] Ceccarelli A, Ballarin M, Montalti M et al (2024) Delirium diagnosis, complication recognition, and treatment knowledge among nurses in an Italian local hospital: a cross-sectional study. Nurs Rep 14:767–776. 10.3390/nursrep14020059 38651471 10.3390/nursrep14020059 PMC11036222

[44] Bail K, Grealish L (2016) ‘Failure to Maintain’: a theoretical proposition for a new quality indicator of nurse care rationing for complex older people in hospital. Int J Nurs Stud 63:146–161. 10.1016/j.ijnurstu.2016.08.001 27658271 10.1016/j.ijnurstu.2016.08.001

