# Supplementary material for: Reasons influencing the nurses’ prioritization process while preventing and managing delirium: findings from a qualitative study
Source: Aging Clin Exp Res. 2024 Aug 26;36(1):178. doi: 10.1007/s40520-024-02818-3 (PMC11347455; doi:10.1007/s40520-024-02818-3)
Supplement: Supplementary file 1 — Supplementary file1 (DOCX 21 KB) [file 40520_2024_2818_MOESM1_ESM.docx]

**SUPPLEMENTARY TABLE 1: Demographic characteristics of the participant’s nurses**

| **Variables** | **N (%)**  **56 (100)** |
| --- | --- |
| **Age,** CI (95%) | 31.6 (29.6–33.6) |
| **Female** | 39 (69.6) |
| **Undergraduate nursing education** |  |
| Bachelor’s degree | 53 (94.6) |
| **Post-graduate education** |  |
| Master’s degree | 12 (21.4) |
| **Continuing education course(s) on delirium** | 15 (26.8) |
| **Work setting** |  |
| Internal medicine | 31 (55.4) |
| Geriatrics | 15 (26.8) |
| Post-acute-intermediate care | 10 (17.8) |
| **In the current unit**  I spent the most time of my professional experience | 38 (67.9) |
| Years of experience, CI (95%) | 4.5 (2.7–6.2) |

**Legend:** CI: confidence interval, N: number of Registered Nurses interviewed
